# Novel inhibitors and activity-based probes targeting serine proteases

**DOI:** 10.3389/fchem.2022.1006618

**Published:** 2022-09-28

**Authors:** Timothy E. G. Ferguson, James A. Reihill, S. Lorraine Martin, Brian Walker

**Affiliations:** Biomolecular Sciences Research Group, School of Pharmacy, Queen’s University Belfast, Belfast, United Kingdom

**Keywords:** serine protease, activity probes, cystic fibrosis, CF, activity-based profiling, inhibitors

## Abstract

Serine proteases play varied and manifold roles in important biological, physiological, and pathological processes. These include viral, bacterial, and parasitic infection, allergic sensitization, tumor invasion, and metastasis. The use of activity-based profiling has been foundational in pinpointing the precise roles of serine proteases across this myriad of processes. A broad range of serine protease-targeted activity-based probe (ABP) chemotypes have been developed and we have recently introduced biotinylated and “clickable” peptides containing P1 *N*-alkyl glycine arginine *N*-hydroxy succinimidyl (NHS) carbamates as ABPs for detection/profiling of trypsin-like serine proteases. This present study provides synthetic details for the preparation of additional examples of this ABP chemotype, which function as potent irreversible inhibitors of their respective target serine protease. We describe their use for the activity-based profiling of a broad range of serine proteases including trypsin, the trypsin-like protease plasmin, chymotrypsin, cathepsin G, and neutrophil elastase (NE), including the profiling of the latter protease in clinical samples obtained from patients with cystic fibrosis.

## Introduction

Serine proteases play varied and manifold roles in important biological, physiological, and pathological processes. These include the combined action of viral and host serine proteases in facilitating cellular entry and replication of viruses such as hepatitis-C, Zika, dengue, and SARS-CoV-2 (Tomei et al., 1993; Tong et al., 1996; Skorenski et al., 2016; Majerova et al., 2019; Muller et al., 2022). Similarly, the combined action of microbial and host serine proteases is known to promote and propagate bacterial infection (Boucher et al., 1996; Jenal and Fuchs, 1998; Small et al., 2013; Kugadas et al., 2016; Wessler et al., 2017). Similarly, parasite serine proteases have long been considered therapeutic targets considering their essential involvement in life-cycle progression and host invasion by these organisms (Salter et al., 2002; Dvorak and Horn, 2018; Leontovyc et al., 2018; Gandhi et al., 2020; Lidumniece et al., 2021). Additionally, several common allergens produced, for example, by house dust mites and cockroaches are serine proteases that initiate mast cell activation/sensitization and cause disruption of epithelial barriers, in a range of human allergic conditions (Montealegre et al., 2004; Arizmendi et al., 2011; Argrawal and Arora, 2018; Li et al., 2019; Reihill et al., 2020). Multiple serine proteases have also been demonstrated to facilitate tumor dissemination/invasion in a range of human cancers (Wilson et al., 2005; Bekes et al., 2011; Deryugina and Quigley, 2012; Pawar et al., 2019; Lovell et al., 2021; Tagirasa and Yoo, 2022).

The use of activity-based profiling has been foundational in pinpointing the precise roles of serine proteases across this myriad of processes. A broad range of serine protease (SP) activity-based probe (ABP) chemotypes have been developed.

**FIGURE 1:**
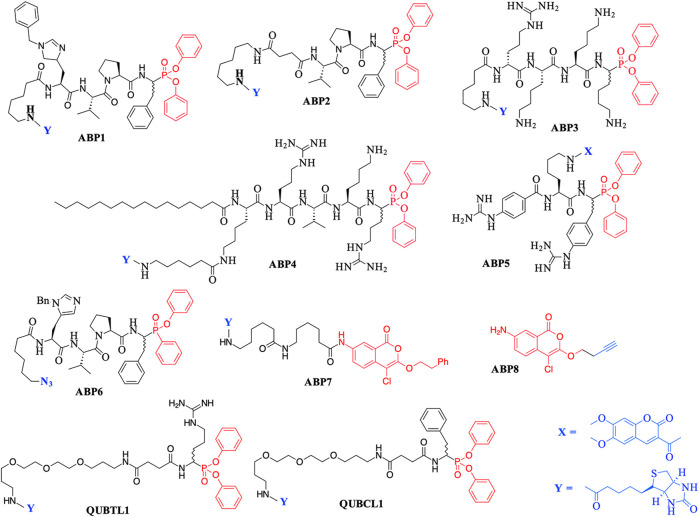
Previously reported Activity Based Probes (ABP) for the serine proteases. Reactive serine-directed electrophiles are shown in red, and reporter groups are shown in blue.

The major chemotype by far consists of peptides containing C-terminal di-aryl phosphonate analogues of amino acids. These include biotinylated, fluorescently labeled, and “click-chemistry” compatible derivatives. For example, biotin derivatives of di-aryl phosphonates have been synthesized for the detection/imaging of lymphocytic and neutrophilic SPs such as elastase, cathepsin G, and proteinase 3 (Abuelyaman et al., 1997; Gilmore et al., 2006; Guarino et al., 2014). The peptides ABP1, ABP2, and QUBCL1, shown in Figure 1, are examples of this chemotype.

The biotinylated di-aryl phosphonates ABP3, ABP4, and QUBTL1 (Figure 1) all contain a C-terminal arginine diphenyl phosphonate, fulfilling the primary (P1) specificity of trypsin-like SPs (nomenclature of Schechter and Berger, 1967). ABP3 was designed to target Zika virus NS2B/NS3 protease (Rut et al., 2017), ABP4 is a selective furin inhibitor (Ferguson et al., 2016), and QUBTL1 functions as a broad-spectrum ABP of trypsin-like proteases including human airways trypsin (HAT), matriptase, and furin (Martin and Walker, 2017). QUBTL1 is cell impermeable, inhibits extracellular trypsin-like proteases, and blocks the proteolytic activation of the epithelial Na^+^ channel (ENaC) in primary cultures of cystic fibrosis (CF) airway cells. Blockade of ENaC activation leads to an improvement in the airway surface liquid (ASL) height and restoration of mucociliary function, both of which are adversely impacted/compromised in CF patients, resulting in mucus plugging and chronic bacterial infection (Reihill et al., 2016). This work has led to the identification of a highly specific, cell-permeable, small molecule, an inhibitor of furin (BOS-318), which also restores ASL height and mucociliary function (Douglas et al., 2022).

An example of a fluorescently labeled di-aryl phosphonate is ABP5. This and additional analogues bearing differing fluorescent groups were developed by Häußler et al. (2016) for the activity profiling of the trypsin-like protease matriptase, iso-forms of which are involved in a range of diseases including pulmonary fibrogenesis, various skin disorders, and cancers (Netzel-Arnett et al., 2006; Chen et al., 2011; Bardou et al., 2016). A library of di-aryl phosphonates bearing a range of fluorescent reporter groups has also been developed for neutrophilic SPs, some of which display exquisite selectivity of action for individual proteases (Kasperkiewicz et al., 2017). A comprehensive review of the synthesis, inhibitory activity, and use of suitably functionalized di-aryl phosphonate as ABPs has been published recently (Máslanka and Muca, 2019).

Peptidyl monophenyl phosphinate ABPs suitable for click-chemistry derivatization such as the azide derivative, ABP6 (see Figure 1), have also been developed recently and employed for imaging serine proteases in live neutrophils (Kahler and Verhelst, 2021). Additional examples are likely to be produced, as previous studies have demonstrated the greater rate of irreversible inhibition of chymotrypsin and neutrophil elastase (NE) by monophenyl phosphinates in comparison to their exact analogous diphenyl phosphonate counterparts (Walker et al., 2000).

Additionally, the use of *iso*-coumarins as serine protease ABPs is also well documented. For example, the biotinylated derivative ABP7 and related analogues have been used for the activity-based profiling of intracellular serine proteases (Kam et al., 1993; Winkler et al., 1996; Haedke et al., 2012), and the alkyne derivative ABP8 was developed as one of a series of “clickable” ABPs to profile the intramembrane located, rhomboid serine proteases (Wolf et al., 2015a; Wolf et al., 2015b).

**FIGURE 2:**
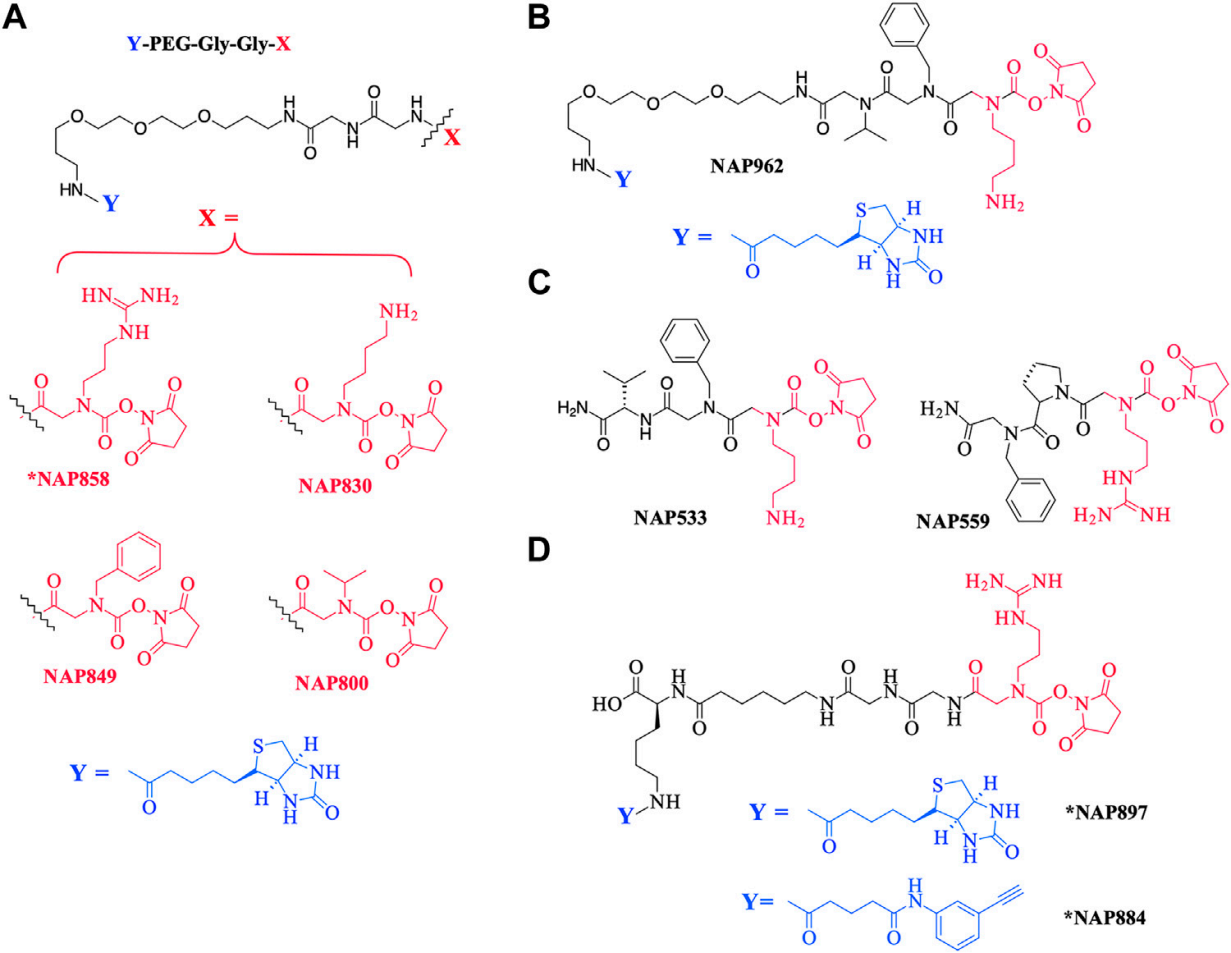
Chemical structures of N-alkyl glycine N-Hydroxy succinimidyl carbamates. **(A)** *NAP858, NAP830, NAP849, and NAP800. **(B)** NAP962. **(C)** NAP533, and NAP559. **(D)** *NAP884 and *NAP897. Structures marked with * are taken from Ferguson et al. (2022), all others are from the present study.

As a further contribution to ABP development for the serine proteases, we have recently introduced biotinylated (NAP987) and “clickable” (NAP884) peptides containing P1 *N*-alkyl glycine arginine carbamates (see Figure 2 for structures) as ABPs for detection/profiling of trypsin-like serine proteases (Ferguson et al., 2022).

This present study details the preparation of additional examples of this ABP chemotype (see Figure 2 for structures), discusses their inhibitory activity and illustrates their use in the detection/disclosure of a broad range of serine proteases including trypsin, chymotrypsin, cathepsin G, neutrophil elastase (NE), the trypsin-like protease plasmin, and demonstrates the use of the NE-directed ABP for the detection of active forms of this protease in samples obtained from patients with cystic fibrosis.

## Materials and methods

### Materials

All reagents and solvents were from Sigma-Aldrich unless otherwise indicated. All standard Fmoc-protected amino acids were supplied by Activotec. The Fmoc-protected *N*-alkyl glycine derivatives Fmoc-*N*Phe-OH and Fmoc-*N*Lys-(N-ε-Boc)-OH were obtained from Polypeptide Labs. Biotin-PEG NovaTag resin was supplied by Merck Millipore. ESI-MS analysis was carried out by ASEPT (Queen’s University Belfast).

Human plasmin and thrombin were supplied by R&D Systems. Human neutrophil elastase (NE) and chymotrypsin were supplied by Calbiochem. Levels of active NE in cystic fibrosis (CF) sol were determined using the ProteaseTag^®^ Active Neutrophil Elastase Immunoassay, obtained from ProAxsis Ltd. The assay was performed exactly according to the manufacturer’s instructions.

SDS-PAGE was carried out using NuPAGE Novex 4–12% Bis-Tris protein gels (1.0 mm) using a PowerEase 500 power supply with a SeeBlue Plus2 pre-stained Protein Standard as a reference ladder, all supplied by Invitrogen. Western blotting was achieved with an X-Cell II Blot Module (Invitrogen) unto an Amersham Hybond ECL Nitrocellulose membrane (GE Healthcare). Luminata Forte Western HRP substrate was supplied by EMD Millipore. Streptavidin-HRP was supplied by Vector Laboratories.

### Synthesis of inhibitors

The chemical structures of the compounds synthesized as part of this present study are illustrated in Figure 2. All compounds were synthesized using solid-phase synthesis (SPS) methods (full details are provided in the Supplementary Material).

**FIGURE 3:**
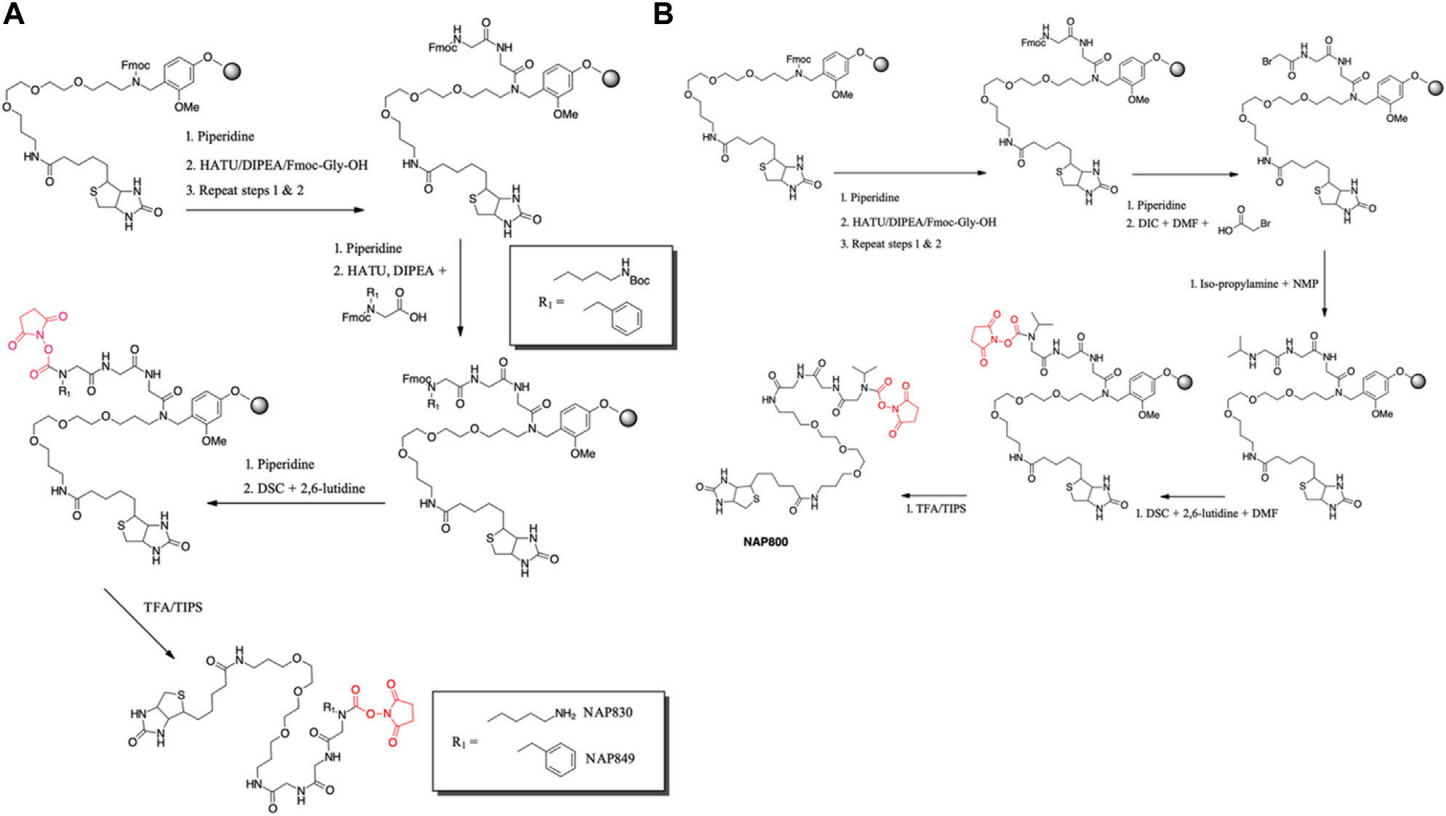
Solid phase synthesis approaches, used in the present study, for the preparation of N-alkyl glycine N-hydroxy succinimidyl carbamates. **(A)** NAP849 andNAP830. **(B)** NAP800.

NAP830 and NAP849 were synthesized utilizing the solid-phase approach employing Biotin-PEG NovaTag resin (NovaBiochem), as shown in Figure 3A. This resin contains a pegylated-diamine residue functionalized with biotin at one amino end, while the other is coupled to the resin beads, through a benzyl linker in the form of a Fmoc-protected 2^o^ amine derivative. For each sequence, the synthesis was commenced by treating samples of the Biotin-PEG NovaTag resin with piperidine to remove the Fmoc-protecting group of the resin-bound 2^o^ amine, followed by two consecutive couplings of Fmoc-Gly-OH, using standard Fmoc-SPS methods (Walker, 1994). The target sequences were then extended/elaborated by the reaction with the corresponding, commercially available, Fmoc-protected *N*-alkyl glycine monomers (Fmoc-*N*Lys-(*N*-e-Boc)-OH for NAP830 and Fmoc-*N*Phe-OH for NAP849), which enabled these residues to be incorporated directly into the common peptidic core sequence, using standard Fmoc-SPS coupling protocols.

Compound NAP962 was synthesized following the same protocol, with the exception that after the unmasking of the Fmoc-protected 2^o^ amine of the biotin-PEG NovaTag resin, the resin was subjected to three cycles of acylation employing, first, Fmoc-*N*Val-OH, followed by Fmoc-*N*Phe-OH and then with Fmoc-*N*Lys-(N-e-Boc)-OH, again using standard Fmoc-SPS methods.

Finally, the Fmoc-protecting group was then removed from each of the terminal *N*-alkyl glycine residues of NAP830, NAP849, and NAP962, prior to the formation of their respective *N*-hydroxy succinimidyl (NHS) carbamate derivative (see below).

Compound NAP800, containing the *N-*alkyl glycine residue *N*Val, was synthesized using the submonomer approach, essentially according to a previously reported method (Tran et al., 2011) (Figure 3B). Consequently, after derivatization of the Biotin-PEG NovaTag resin with the two glycine residues (following the same protocols outlined for NAP830, NAP849, NAP962, and NAP974), the terminal glycine residue was bromoacetylated, in a di-isopropyl carbodiimide (DIC) mediated reaction, using a ten-fold excess of bromoacetic acid. This coupling was repeated (4 × 30 min) with fresh reagents until the Kaiser test (Kaiser et al., 1970) indicated complete acylation. This was followed by the addition of a solution of isopropylamine in *N*-methylpyrrolidone (NMP) to allow displacement of the bromine to form the *N*Val alkyl glycine residue.

The formation of the *N*-hydroxy succinimidyl (NHS) carbamate derivative of all the sequences was then achieved essentially according to a previously reported method (Niphakis et al., 2013). Briefly, a solution containing *N, N*′-disuccinimidyl carbonate (DSC) and 2,6-lutidine in anhydrous DCM/DMF (1:1) was added to the deprotected resin until chloranil analysis indicated a complete reaction of the 2 amine function.

The un-biotinylated sequences NAP559 and NAP533 were synthesized on an MBHA Rink amide resin, using essentially the same standard Fmoc-SPS methods outlined above. The synthesis of NAP559 was achieved using the sequential coupling/deprotection of Fmoc-*N*Phe-OH, Fmoc-Pro-OH, and Fmoc-*N*Arg-(Pbf)-OH. In a similar manner, NAP533 was prepared using sequential coupling/deprotection of Fmoc-Val-OH, Fmoc-*N*Phe-OH, and Fmoc-*N*Lys-(N-e-Boc)-OH. The Fmoc-protecting group was then removed from the terminal *N*-alkyl glycine residue of each sequence, prior to the formation of their respective NHS carbamate derivative, as outlined above.

Subsequent TFA-mediated cleavage of each of the target *N*-alkyl carbamate-containing sequences from the resin beads (and deprotection of the Boc-group and Pbf-group from NAP533/NAP962 and NAP559, respectively) was achieved using TFA/TIPS/DCM (95:2.5:2.5), and the final products were obtained following the standard workup and precipitation in diethyl ether. HPLC analysis of final compounds was carried out on an Agilent Technologies 1,260 Infinity machine using a Waters Atlantis C18 5 μm, 4.6 Å∼ 150 m column. A two-phase solvent system was used: A) 0.05% (v/v) TFA in water and B) 0.05% (v/v) TFA in acetonitrile. A linear gradient elution system was implemented at 1 ml/min from 0% B) to 90% B) over 45 min, held for a further 10 min, then back to 0% B) over 10 min, and held for a final 10 min. A UV detector was used to monitor absorbance at *λ* = 216 nm.

All synthesized inhibitors were analyzed by ESI-MS and gave satisfactory data consistent with their proposed structures (see the Supplementary Material).

### Inhibition studies

A range of inhibitor concentrations were prepared from a 10 mM stock solution of the test compounds (NAP533, NAP559, NAP800, NAP830, NAP849, and NAP962) in *N, N*-dimethylformamide (DMF). Recombinant protease activities were assayed using the cognate fluorogenic substrates as follows: trypsin (Z-Gly-Gly-Arg-NHMec), thrombin (Boc-Val-Pro-Arg-NHMec), plasmin (Boc-Val-Leu-Lys-NHMec), human neutrophil elastase (MeO-Succinyl-Ala-Ala-Pro-Val-NHMec), and chymotrypsin (Succinyl-Ala-Ala-Pro-Phe-NHMec). Each substrate was used at a final concentration of 50 μM, in the appropriate buffer for each protease. All inhibition assays were performed in microtiter plates maintained at 37°C in a final volume of 100 μL. The reaction was initiated by the addition of each protease (approx. 0.01 μg/well) and the rate of substrate hydrolysis was recorded continuously at 37°C, over a 60 min period, by measuring the rate of increase in fluorescence (at λex 360 nm, λem 480 nm) using a FLUOstar Optima microplate reader (BMG Labtech).

The resultant inhibition progress curves obtained for each inhibitor were analyzed by the kinetic models developed by Tian and Tsou (1982) and Walker and Elmore (1984), for irreversible inhibitors, with curve fitting being achieved using GraFit (Erithacus Software), as described previously (Ferguson et al., 2016).

### Molecular modeling studies

The X-ray coordinates of human thrombin in complex with the chloromethyl ketone inhibitor D-Phe-Pro-Arg-CH_2_Cl and human plasmin in complex with an analogue of sunflower seed trypsin inhibitor were extracted from Protein Data Bank (PDB) files 1PPB (Bode et al., 1989) and 6D40 (Swedberg et al., 2019), respectively. SDF files of NAP533 and NAP559 were generated using ChemDoodle^®^ v11.0 (iChemLabs). Protein preparation (solvation and charge assignment), ligand preparation, and molecular docking of NAP559 with thrombin, and NAP533 with plasmin, were performed using Molecular Forecaster software (Therrien et al., 2012; Moitessier et al., 2016). Visualization of molecular structures was achieved using PyMol™ version 2.4.0 (Schrodinger, LLC).

### Evaluation of compounds as activity-based probes

Samples of recombinant protease (trypsin, plasmin, thrombin, chymotrypsin, or HNE, as indicated in the relevant figure legends) were treated with each of the newly synthesized compounds at a concentration of 50 μM, for 30 min, at 37°C. A control sample (no ABP) was prepared by treating the recombinant protease with solvent vehicle (DMF) only, used to prepare the stock solution of each ABP. Samples were also pre-treated (±90°C for 30 min) before being incubated with each putative ABP. With trypsin and thrombin, further samples were prepared where Z-Arg^P^-(OPh)_2_ or NAP559, respectively, was added 30 min prior to the addition of the ABP under examination. All treated samples were then denatured with SDS containing reducing treatment buffer (10 min at 95°C), followed by resolution by SDS-PAGE and subjected to Western blotting. Resolved proteins were then transferred onto the nitrocellulose membrane and labeled with streptavidin-HRP. The ABP–protease complex was then visualized following treatment with Luminata Forte HRP substrate.

## Results

The precise chemical structures of the putative *N*-alkyl glycine-based NHS-carbamate activity-based probes, synthesized in the present study, are illustrated in Figures 2A,B; they are described also in US Patent Nos. 11, 104, and 703 (issued 31 August 2021).

NAP849 was designed as an ABP for chymotrypsin-like proteases, NAP800 was designed as a neutrophil elastase ABP, and NAP830 as an ABP for trypsin-like proteases. The incorporation of the two glycine residues interspersed between the biotinylated-PEG motif and each of the P1 *N*-alkyl glycine NHS-carbamates, is a common feature of NAP800, NAP830, and NAP849. This was to ensure the optimal reaction of each ABP with its target SP and to enable the subsequent detection of the respective SP–ABP complexes by streptavidin-HRP, through the interaction with the biotin grouping.

NAP962 was designed as an ABP for plasmin, a trypsin-like serine protease with known roles in tissue remodeling during carcinogenesis (Deryugina and Quigley, 2012; Bharadwaj et al., 2021; Heissig et al., 2021) and the activation of renal ENaC, which is implicated in the etiology of pre-eclampsia (Passero and Mueller, 2008; Buhl and Ulla, 2012). NAP962 contains *N*-alkyl benzyl and *N*-alkyl iso-propyl groups as mimetics, respectively, of P2 (phenylalanyl) and P3 (valyl) side-chain residues that occur in known nitrile and aldehyde inhibitors of plasmin (Swedberg and Harris, 2011; Teno et., 2012). These P2 and P3 *N*-alkyl-glycine residues replace the glycyl-glycine dipeptide motif in NAP830. In common with NAP830, NAP962 contains an *N*Lys-residue, a mimetic of the amino acid lysine, which is often found at the P1 position of peptide and protein substrates for plasmin (Hervio et al., 2000).

Figure 2C also shows the structures of two non-biotinylated *N*-alkyl glycine carbamates NAP533 and NAP559, designed to function against plasmin and thrombin, respectively. These were synthesized as potential blocking compounds for use in activity-based profiling applications to establish the fidelity of ABP disclosure of SP targets. NAP533 contains the same P2 and P3 recognition elements that are present in the biotinylated compound NAP962. NAP559 incorporates P2 (prolyl) and P3 *N*-alkyl benzyl residues mirroring the P2-proline and P3-phenylalanine residues in known chloromethyl ketone (Kettner and Shaw, 1979; Bode et al., 1989), aldehyde (Krishnan et al., 1998), and boronic acid (Webber et al., 1995) inhibitors of thrombin.

### Inhibitory evaluation of compounds

**FIGURE 4:**
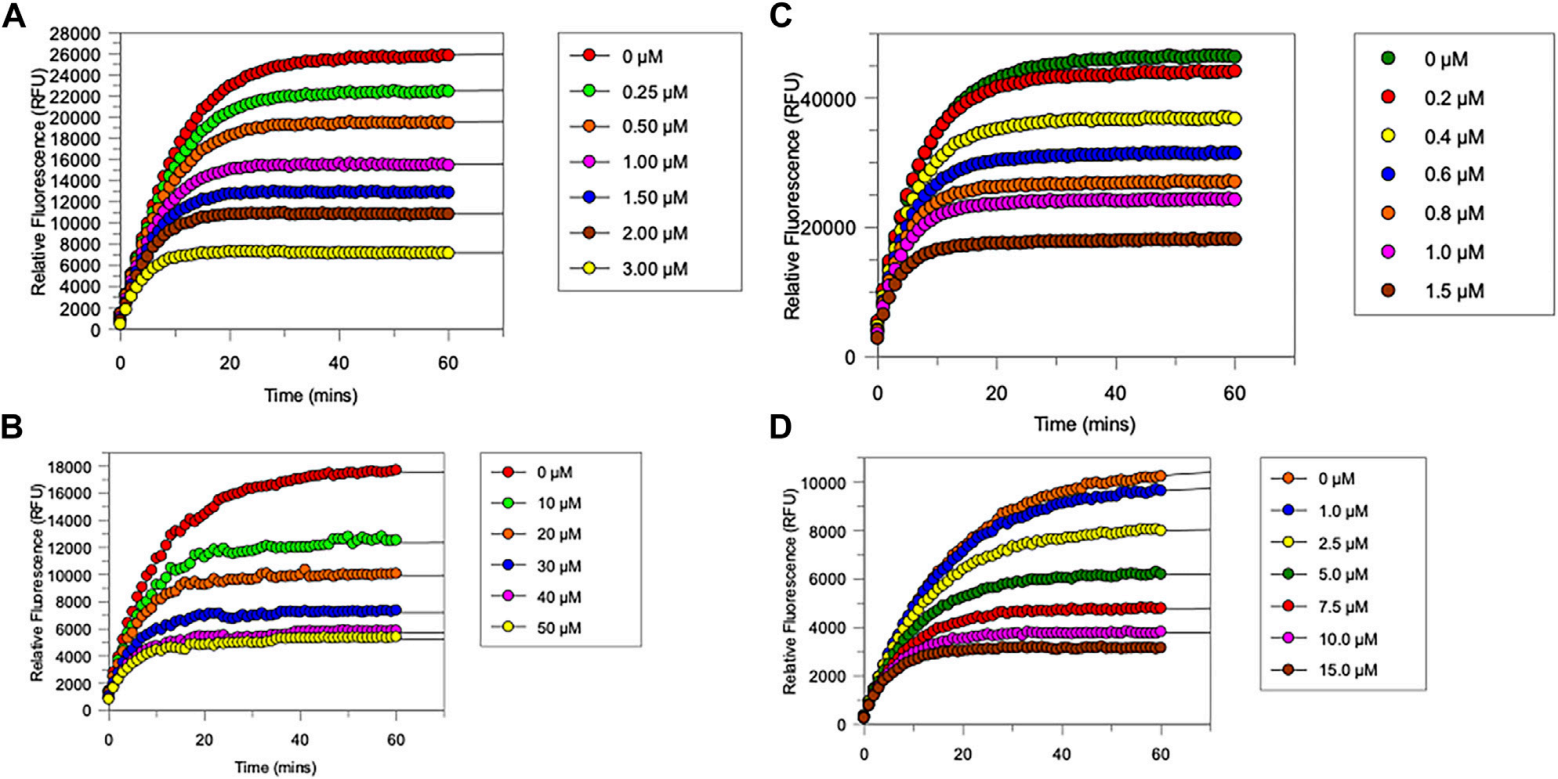
Progress curves for **(A)**, chymotrypsin-catalysed hydrolysis of Succinyl-Ala-Ala-Pro-Phe-NHMec, in the presence of varying concentrations of NAP849. **(B)**, NE-catalysed hydrolysis of MeO-succinyl-Ala-Ala-Pro-Val-NHMec, in the presence of varying concentrations of NAP800. **(C)** Trypsin-catalysed of Z-Gly-Gly-Arg-NHMec, in the presence of NAP830. **(D)** Plasmin-catalysed hydrolysis of Boc-Val-Leu-Lys-NHMec, in the presence of varying concentrations of NAP962. Assays were carried out in PBS, pH 7.4, at 37°C, in the presence of a range of inhibitor concentrations (0.25–50 μM) and a fixed concentration (50 μM) of the appropriate substrate for each protease.

**FIGURE 5:**
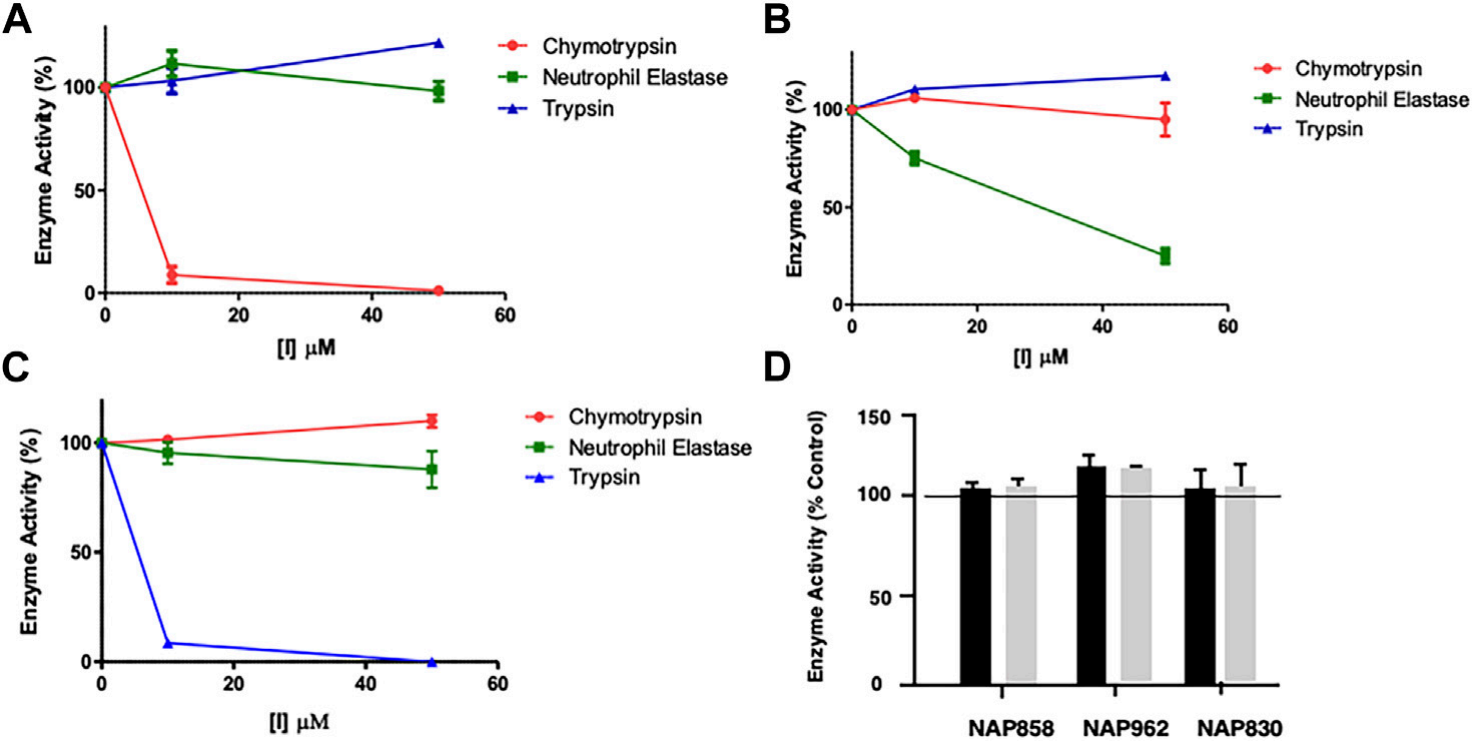
Results of “off-target“ studies. NAP849 **(A)**, NAP800 **(B)** and NAP830 **(C)** were tested against trypsin, chymotrypsin, and NE. Samples (approx. 0.01 μg) of each protease were incubated with the indicated concentrations of test compound for 2 min, in PBS, pH 7.4, at 37°C. **(D)** NAP858, NAP962 and NAP849 were tested against cathepsin B at the indicated concentrations in 0.1 M MES, pH 6.1, containing 1 mM EDTA, for 2 min, at 37°C. Aliquots (approx. 10 μL) of each incubation sample were then added to a fresh sample (990 μL) of PBS for the serine proteases and MES for cathepsin B, containing the appropriate substrate for each protease, to measure their residual activity. Controls incubations were performed in the absence of test compound, but in the presence of an appropriate volume of DMF vehicle used to prepare stock solutions of each inhibitor under study.

All newly synthesized compounds were evaluated for their ability to inhibit their intended target protease, in the presence of competing substrates; the results of these studies are shown in Figure 4. “Off-target” studies were also performed for each peptide against the non-target proteases. The results of these studies are shown in Figure 5 and are discussed below.

**FIGURE 6:**
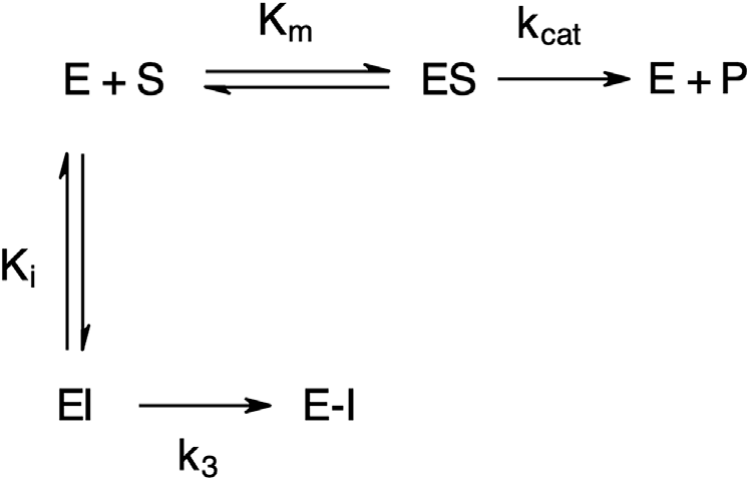
Scheme for irreversible inhibition. E, Enzyme; S, Substrate; ES, Enzyme-Substrate complex; P, Product; EI, Enzyme-Inhibitor complex; Km, Michaelis-Menten constant; *K*_i_, Inhibition constant; *k*_cat_, catalytic constant; *k*_3_, first-order rate constant; E−I, irreversible enzyme-inhibitor complex.

The “product progress” curves for substrate hydrolysis in the presence of an inhibitor (Figure 4) are indicative of the action of irreversible inhibitors operating through the kinetic scheme illustrated in Figure 6. In this scheme, the formation of the reversible enzyme–inhibitor complex (EI) is zcharacterized by the inhibitor constant *K*_i_. This is converted into the covalent/irreversible complex (E-I) characterized by a first-order rate constant *k*_3_. The overall second-order rate constant for the inactivation of E by I is given by the ratio *k*_3_/*K*_i_ (Walker and Elmore, 1984; Tsou, 1988).

As anticipated, NAP800 exhibited potent inhibition of NE, as evidenced by the “progress curves” for the formation of 7-amino-4-methyl coumarin (NH_2_Mec) generated by the protease-catalyzed hydrolysis of MeO-succinyl-Ala-Ala-Pro-Val-NHMec, in the presence of this inhibitor (Figure 4A). The determined second-order rate constant (*k*_3_/*K*_i_) for the inactivation of NE by this *N*-alkyl carbamate was 6.41 × 10^4^ M^−1^ min^−1^ (Table 1) is approximately five-fold greater than the second-order rate constant reported for the exactly analogous valine diphenyl phosphonate analogue QUBEL1 (biotin-PEG-succinyl-Val^P^-(OPh)_2_) (Gilmore et al., 2006).

**TABLE 1:** Kinetic constants for the inhibition of NE, chymotrypsin, and trypsin, by various ABPs.

Protease	Inhibitor	*K*_i_ (M)	*k*_3_ (min^−1^)	*k*_3_ / *K*_i_ (M^−1^ min^−1^)
Chymotrypsin	NAP849 (*n*=3)	8.9 (± 0.7) × 10^−7^	0.85 (± 0.29)	1.28 (± 0.44) × 10^6^
**“**	*QUBCL1	1.08 × 10^−6^	0.119	1.11 × 10^5^
NE	NAP800	6.0 (± 1.4) × 10^−6^	0.38(± 0.06)	6.4 (± 0.57) × 10^4^
**“**	*QUBEL1	-	-	1.29 × 10^4^
Trypsin	NAP830	2.2 (+ 0.8) × 10^−7^	0.6 (+ 0.2)	2.7 (+ 0.5) × 10^6^
“	**NAP858	2.3 (+ 1.3) × 10^−7^	0.5 (+ 0.1)	2.1 × 10^6^
“	***QUBTL2			1.1 × 10^6^

Values are shown as Mean ± SD (*n*=2). * Gilmore et al. (2009). ** Ferguson et al. (2022). *** Martin and Walker (2017).

Similarly, NAP849 exhibited potent inhibition of chymotrypsin with rapid, time-dependent saturation of the protease being achieved (Figure 4B). This phenylalanine analogue exhibited an excellent second-order rate constant for the inactivation of chymotrypsin of 1.28 × 10^6^ M^−1^ min^−1^ (see Table 1), which is approximately ten-fold greater than the second-order rate constant reported for the exactly analogous phenylalanine diphenyl phosphonate analogue biotin-PEG-succinyl-Phe^P^-(OPh)_2_ (structure QUBCL1 in Figure 1) (Gilmore et al., 2006).

NAP830 displayed potent irreversible inhibition of trypsin (Figure 4C). A second-order rate constant of 2.74 × 10^6^ M^−1^ min^−1^ was determined for the inactivation of trypsin by NAP830 (Table 1). This is very close to the value obtained (2.06 × 10^6^ M^−1^ min^−1^) for its exactly analogous *N*-alkyl arginine counterpart NAP858, against the same protease, previously reported by us (Ferguson et al., 2022). This equipotency of the two ABPs was anticipated since trypsin is known to cleave peptides and proteins equally efficiently at lysine and arginine residues (Evnin et al., 1990). In keeping with both NAP800 and NAP849, NAP830 also exhibited two-fold greater effectiveness as an irreversible inhibitor of trypsin than its directly analogous lysine diphenyl phosphonate counterpart Biotin-PEG-Succinyl-Lys^P^-(OPh)_2_ (QUBTL2), previously reported as an ABP for trypsin-like serine proteases (Martin and Walker, 2017).

NAP962 was designed to function as an inhibitor of plasmin. Satisfyingly, it exhibited potent time-dependent irreversible inhibition of this protease (Figure 4D). A comparison of the kinetic inhibition data for this carbamate and NAP830 against plasmin, along with the previously reported broad-spectrum ABP QUBTL1 for trypsin-like enzymes, are summarized in Table 2. Based on their respective second-order rate constants, NAP962 displayed a higher potency against plasmin than the broad-spectrum probe for trypsin-like proteases (NAP830). The increased effectiveness of NAP962 in comparison to NAP830 against plasmin is due to improvements in *K*_i_ and *k*_3_ of the former. This is likely due to the incorporation of P3 and P2 recognition elements of known nitrile and aldehyde plasmin inhibitors (Swedberg and Harris, 2011) into NAP962, as opposed to the glycyl-glycine dipeptide unit in NAP830. The un-biotinylated carbamate sequence NAP533 was also found to display irreversible inhibition of plasmin. Furthermore, the potency of NAP533 against plasmin is very similar to that observed with NAP962. This would suggest that the replacement of the *N*Val residue in the biotin-containing sequence with the natural *S*-amino acid valine does not have a detrimental effect on potency, despite the side chain of the former residing in a position that lies between the stereochemistry of *R*- and *S*-amino acids (Simon et al., 1990).

**TABLE 2:** Kinetic constants for the inhibition of plasmin by various ABPs.

Inhibitor	*K*_i_ (M)	*k*_3_ (min^−1^)	*k*_3_ / *K*_i_ (M^−1^ min^−1^)
NAP830	7.2 (± 0.3) × 10^−6^	0.4 (± 0.05)	6.2 (± 0.9) × 10^4^
NAP962	4.2 (± 0.2) × 10^−6^	0.6 (± 0.1)	1.4 (± 0.3) × 10^5^
NAP533	2.2 (± 0.2) × 10^−6^	0.4 (± 0.04)	1.7 (± 0.3) × 10^5^
*QUBTL1	8.9 (± 2.0) × 10^−8^	0.3 (± 0.05)	3.1 (± 0.2) × 10^6^

Values are shown as Mean ± SD (*n*=2). * Martin and Walker (2017).

Interestingly, the broad-spectrum ABP for trypsin-like SPs, biotin-PEG-succinyl-arginine diphenyl phosphonate QUBTL1 (Reihill et al., 2016) displayed the highest second-order rate constant out of all three probes tested against plasmin. However, it should be noted it also displayed the lowest first-order rate constant (*k*_3_) of the three ABPs tested against this protease, suggesting the slowest rate of formation of the covalent complex between this protease and inhibitor.

Finally, the un-biotinylated sequence NAP559 was also found to display potent irreversible inhibition of thrombin, for which it was designed to act against (see Supplementary Table S1). This was reassuring since this peptide is modeled directly on peptide arginine chloromethyl ketones containing P3 phenylalanine and P2 proline, which are very effective thrombin inhibitors (Kettner and Shaw, 1979; Walker et al., 1985). NAP559 was designed as a potential blocking compound for use in activity-based profiling applications of thrombin (see below).

### Evaluation of “off-target” inhibitory effects

Two elements were investigated regarding possible “off-target” effects–determining the extent of cross-reactivity of each of the new putative ABPs between the various serine protease family members and assessing their collateral inhibitory action against another mechanistic class–the cysteine proteases, using cathepsin B (CTSB) as an exemplar. The results of these studies are presented in Figure 5.

As mentioned previously, NAP849 displayed potent irreversible inhibition of chymotrypsin, which was in direct contrast to a complete absence of inhibition against trypsin and neutrophil elastase (Figure 5A), indicating the highly selective nature of this compound for chymotrypsin-like proteases. Similarly, the NE-directed sequences NAP800 exhibited no “off-target” or collateral inhibition against the other two serine proteases tested (Figure 5B). NAP830, which contains an *N*Lys alkyl glycine carbamate at P1 and was designed to target trypsin-like SPs, exhibited no “off-target” effects when tested against chymotrypsin and neutrophil elastase at concentrations up to 50 μM (Figure 5C).

Similarly, the un-biotinylated sequences NAP553 and NAP559, which were designed to be irreversible inhibitors of plasmin and thrombin, respectively, caused only minimal inhibition (<5% of control) against NE and chymotrypsin, and only at the highest concentration (50 μM) studied (see Supplementary Figure S1).

Since the cysteine protease cathepsin B (CTSB) is known to be potently inhibited by various peptide thiomethly ketone derivatives containing P1 lysine or arginine, with some examples exhibiting *K*_i_ values in the low nanomolar range (Wood et al., 2003), we considered it judicious to examine for possible “off-target” effects of NAP830 and NAP962 against this protease, as they both contain a P1 *N*Lys residue. We also included the carbamate NAP858 in this study, as it contains a P1 *N*Arg residue. It was gratifying to find that none of the three carbamates tested exhibited any inhibitory activity against CTSB, when tested at concentrations of 25 and 50 μM (Figure 5D).

### Activity-based profiling of recombinant/purified proteases

Encouraged by the positive inhibition studies performed on each of the newly synthesized carbamate derivatives regarding their potency and selectivity of action, we next evaluated their effectiveness as ABPs against their cognate serine protease.

**FIGURE 7:**
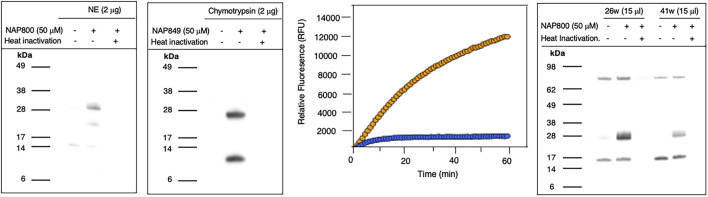
Activity Based Profiling of serine proteases by peptide N-Alkyl glycine carbamates. **(A,B)** record the results for the incubation of NE with NAP800 and chymotrypsin with NAP849. NE and Chymotrypsin were treated (±) with NAP800 and NAP849, respectively (± pre-heat inactivation at 95°C, for 10 min), for 30 min, at 37°C. **(C)** Determination of NE levels in CF samples using fluorogenic substrate assay. NE assays were carried out in PBS, pH 7.4, at 37°C. CF Sol (sample 26w) was used at a final concentration of 2.5% (v/v), and the level of NE contained therein was measured using Succinyl-Ala-Ala-Pro-Val-NHMec (50 μM), in the absence (-- o --) and presence (-- o --) of NAP800 (50 μM). **(D)** Activity based profiling of CF Sol using NAP800. CF Sol samples 26w and 41w were treated (+) with 50 μM NAP800 (+ pre-heat inactivation at 95°C, for 10 min), for 30 min, at 37°C.

NAP800 was evaluated for its ability to act as an ABP of neutrophil elastase. It can be seen from Figure 7A (lane2) that NAP800 labels a protein band corresponding to the expected molecular size/mass of NE (∼29 kDa). Additionally, as expected, heat inactivation of NE prior to incubation with NAP800 resulted in the complete extinguishing of labeling (lane 3), thus indicating that the interaction of this ABP is dependent on an active protease being present and is not due to non-specific chemical modification at some solvent exposed nucleophilic residue on the protease.

NAP849 was next tested for its ability to function as an ABP for chymotrypsin. The results are shown in Figure 7B. This enabled detection of a strong band corresponding to the molecular weight of chymotrypsin (25 kDa), confirming the irreversible inhibitory action of NFP849. An additional lower band was also observed, which is presumed to be a cleaved fragment containing the labeled active site of chymotrypsin. Heat inactivation of the protease prior to incubation with NAP849 completely blocked the incorporation of the ABP (lane 3, Figure 7B).

**FIGURE 8:**
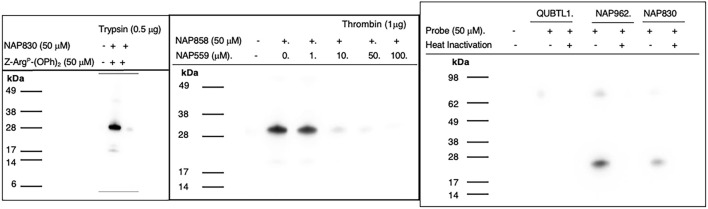
Activity Based Profiling of Trypsin-like serine proteases. **(A)** trypsin was treated (±) with NAP830, for 30 min, at 37°C (± pretreatment with Z-Arg^P^-(OPh)_2_). **(B)** thrombin (1 εg) was pre-treated with various concentrations of NAP559 (30 min, at 37°C) followed by treatment (±) with NAP858 for 30 min, at 37°C. **(C)** Plasmin was treated in turn with QUBTL1, NAP962 and NAP830, (± pre-heat inactivation at 95°C, for 10 min) for 30 min, at 37°C.

NAP830 proved to be an effective ABP for trypsin and enabled the active site labeling and detection of the protease, revealing a band of apparent molecular weight of 23 kDa, following SDS PAGE and western blotting, after incubation of probe and protease (Figure 8A). Pre-treatment of protease with a non-biotinylated, irreversible, active-site directed inhibitor, Z-Arg^P^-(OPh)_2_ (Martin and Walker, 2017) resulted in a quenching/diminution in the signal detected by NAP830 (lane 2, Figure 8A), indicating that this probe is active-site directed and does not cause non-specific chemical modification of the protease.

We reported previously that NAP858 functions as an ABP for thrombin, permitting the detection of a band with an apparent molecular mass of 31 kDa, corresponding to the heavy (catalytic) chain of the protease (Ferguson et al., 2022). This banding pattern is recapitulated in Figure 8B. As mentioned above, we developed NAP559 as a potential ABP blocking inhibitor of thrombin. Thus, it was gratifying to find that when thrombin was pre-treated with NAP559, even at the lowest concentration of 1 μM, there was slight but a discernible diminution in the intensity of the band at 31 kDa, disclosed by NAP858. This band is almost completely extinguished when the protease is pre-incubated with concentrations of 10 and 50 μM of NAP559 (Figure 8B). These observations are explicable based on the second-order rate constant obtained for the inhibition of thrombin by NAP559 (see Supplementary Table S1). Additionally, this labeling study shows that the incorporation of extended residues within NHS carbamate *N-*alkyl glycine-based inhibitors allows potent irreversible inhibition of thrombin and is a potential route for the creation of more potent and selective ABPs for this protease.

We next evaluated the ability of QUBTL1, NAP962, and NAP830 to function as ABPs for plasmin. Samples of the protease incubated, in turn, by all three compounds (all used at 50 μM) were then subjected to the standard SDS-PAGE and western blotting regimens (Figure 8C). A band corresponding to the light (catalytic) chain of plasmin (26 kDa) was visible with samples treated with NAP962 and NAP830. A more intense band was observed using NAP962, which could reflect its higher inhibitory potency (larger *k*_3_/*K*_i_ value) compared to NAP830, revealed by the inhibition studies discussed previously. Heat inactivation of plasmin prior to incubation with the probes resulted in no bands being disclosed, again supporting the contention that the inhibitors function as true ABPs, reacting only with the active protease.

Most surprisingly, QUBTL1 failed to detect any plasmin band, despite exhibiting the highest potency of inactivation of the protease of the three ABPs studied, as adjudged by its second-order rate constant. Failure to detect plasmin, using this compound, was consistent, despite numerous repeated labeling experiments being conducted.

### Activity-based profiling of biological samples

Having demonstrated the successful application of the *N*-alkyl glycine NHS carbamates as ABPs for the labeling of recombinant/purified serine proteases, it was decided to assess their usefulness for disclosing/detecting these enzymes in more complex systems such as biological samples. As an initial test, the valine carbamate analog NAP800, which proved to be a very effective ABP for NE, was investigated for the disclosure/detection of elastase-like proteases in Cystic Fibrosis (CF) Sol samples. NE is the major protease found within the airways of CF patients, which contributes significantly to the chronic lung damage observed in these patients (Amitani, et al., 1991; Caldwell et al., 2005; Dubois et al., 2012; Gifford and Chalmers, 2014; Voynow and Shinbash 2021). Therefore, it was anticipated that active HNE would be present in CF Sol samples and would be ‘revealed’ by incubation with the NAP800 ABP.

Initially, as a quick screen, a fluorogenic enzyme assay was performed on CF Sol samples, using the elastase fluorogenic substrate MeO-Succinyl-Ala-Ala-Pro-Val-NHMec, the results of one such experiment (performed on sample 26w) is recorded in Figure 7C. This assay revealed the presence of an elastase-like activity since there was pronounced substrate cleavage, over a 60 min period. The inclusion of NAP800 in the assay buffer (used at a final concentration of 50 μM) caused a progressive and ultimately complete blockade of substrate cleavage within the CF Sol sample.

Having demonstrated that NAP800 was able to extinguish the elastase-like activity in the CF Sol samples, it was decided to utilize this biotinylated ABP to profile for elastase-like proteases, in these samples. This resulted in the detection of a diffuse band in two CF Sol samples (26w and 41w), which corresponded to the published molecular mass/size (_∼_29 kDa) of neutrophil elastase (Figure 7D). Some additional bands are visible, which can be assumed to be endogenously biotinylated proteins since they are present even in the ‘unprobed’ control samples.

Additionally, there are clear differences in the intensity of the elastase bands between the two Sol samples. For example, CF Sol sample 26w produced a stronger ‘elastase signal’ suggesting higher levels of active protease than the amount present in sample 41w. This corresponds to the amount of active NE determined to be present in these samples using an activity-based immunoassay (ABI), which measures active NE exclusively (Martin et al., 2011; Moffitt et al., 2015). Sample 26w was shown to contain 100.46 μg/ml of active NE, in contrast to 1.66 μg/ml in sample 41w, using this ABI. This variation explains the difference in the band intensity observed in Figure 7D. Such variations in NE levels between patients have been reported previously and can be attributable to some individuals having suffered periods of exacerbation in cystic fibrosis and Bronchiectasis (Griese et al., 2008; Chalmers et al., 2017).

### Covalent docking of NAP558 and NAP533

To provide a further understanding of the binding of the NHS-carbamates to the active sites of their target SPs, we performed *in silico* covalent docking of NAP558 and NAP533 with thrombin and plasmin, respectively. We used these compounds rather than the biotinylated and pegylated ABPs to reduce the computational time required to conduct the *in silico* docking due to the highly mobile/flexible nature of the biotin-PEG motif. These modeling studies were performed using Molecular Forecaster software (Therrien et al., 2012; Moitessier et al., 2016), which permits the *in-silico* covalent docking of a molecule with an enzyme (of known X-ray structure), provided the software can identify/recognize an electrophilic group in the former. This software correctly identified NAP558 and NAP533 as activated carbamates and performed *in silico* covalent docking to estimate the best poses (lowest energy) for their binding to thrombin and plasmin, respectively.

**FIGURE 9:**
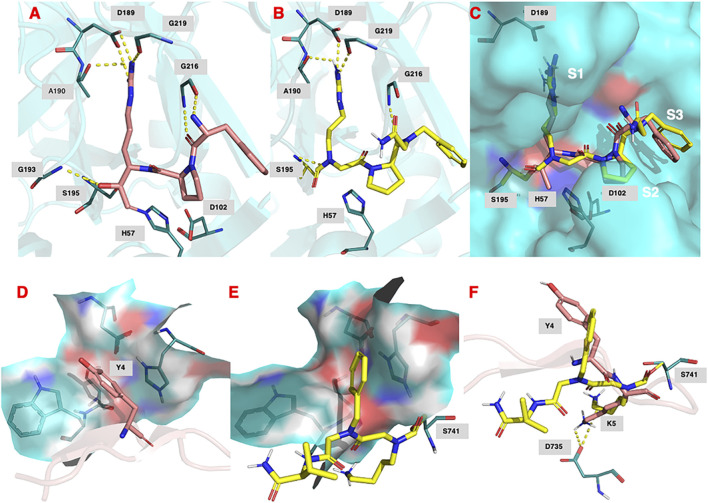
**(A)** Binding of (D)-Phe-Pro-Arg-CH_2_Cl (shown in salmon) within the active site of human thrombin (PDB 1ppb). **(B)** Predicted docking pose and polar interactions of NAP559. NAP559 is predicted to form an acyl enzyme with the active site serine (S195) of thrombin. **(C)** Engagement of thrombin S1, S2 and S3 binding pockets by NAP559 (shown in yellow) and (D)-Phe-Pro-Arg-CH_2_Cl (shown in salmon). **(D)** Binding of SFTI analogue (shown in salmon) within the active site of plasmin (PDB 6d40) highlighting the S2 binding pocket (residues W761, S760, D646 and H603). **(E)** Predicted docking pose of NAP533 (shown in yellow) within active site of plasmin (6d40) highlighting the S2 binding pocket. NAP533 is predicted to form an acyl enzyme with the active site serine (S741 – equivalent to S195 in thrombin). **(F)** Overlay of NAP530 (shown in yellow) and SFTI analogue (shown in salmon).

Figure 9A shows the binding of the chloromethyl ketone ligand D-Phe-Pro-Arg-CH2Cl determined from the X-ray crystallographic structure (PDB 1PPB) of thrombin inhibited by this inhibitor (Bode et al., 1989). Figure 9B shows the remarkable similarity, with D-Phe-Pro-Arg-CH2Cl, in the predicted (lowest energy) docking pose for NAP559 in the active site of thrombin. NAP559 is predicted to engage with the S3, S2, and S1 binding pockets of thrombin, exactly as determined for the peptidyl chloromethyl ketone (Figure 9C). Moreover, it is predicted to form a carbamoylated (acyl) enzyme with the active site serine (S195) of thrombin and engage in multiple polar contacts that share similarities with those determined/established for D-Phe-Pro-Arg-CH_2_Cl, including salt-bridge formation between the guanidino groups of each inhibitor with Asp189 located at the base of the S1 pocket of the protease (compare panel **A** and panel **B**).

Figure 9D shows the binding of an analogue of sunflower seed trypsin inhibitor with human plasmin determined from the X-ray crystallographic structure (PDB 6d40) (Swedberg et al., 2019) and highlights the engagement of the Y4 residue of SFTI with the S2 binding pocket of plasmin (formed by residues W761, S760, D646, and H603), the major subsite determinant for this protease (Hervio et al., 2000; Swedberg and Harris, 2011; Teno et., 2011). Figure 9E, shows the predicted (lowest energy) docking pose for NAP533, and illustrates the similar engagement of the P2 *N*Phe alkyl glycine residue of NAP533 with the S2 binding pocket of plasmin. Figure 9F shows the overlap in the proposed docking pose for NAP533 (shown in yellow) with that determined experimentally by the sunflower seed trypsin inhibitor (shown in salmon). For reasons of clarity, only the Y4 and K5 residues (corresponding to the P2 and P1 residues) of the sunflower seed trypsin inhibitor (SFTI) are shown in this figure. The remainder of the SFTI structure is shown in cartoon form as a ribbon. NAP530 is also predicted to form a carbamoylated (acyl) enzyme with the active site serine S741 of plasmin (equivalent to S195 in thrombin) and to engage in multiple polar contacts that share similarities with those determined for SFTI, including salt-bridge formation between the side-chain primary amino function of the P1 residue of each inhibitor (NLys in NAP533 and Lys in SFTI) with D735 of plasmin (corresponding to D189 in thrombin).

## Discussion

Previously published work from our group has demonstrated that peptide NHS-carbamates containing a P1 *N*-alkyl glycine mimetic of arginine are effective ABPs for trypsin-like serine proteases (Ferguson et al., 2022). This present study has demonstrated that the *N*-alkyl glycine NHS-carbamate chemotype can be expanded to provide additional ABP examples that target a broader range of serine protease subtypes having a P1 specificity other than arginine.

**FIGURE 10:**
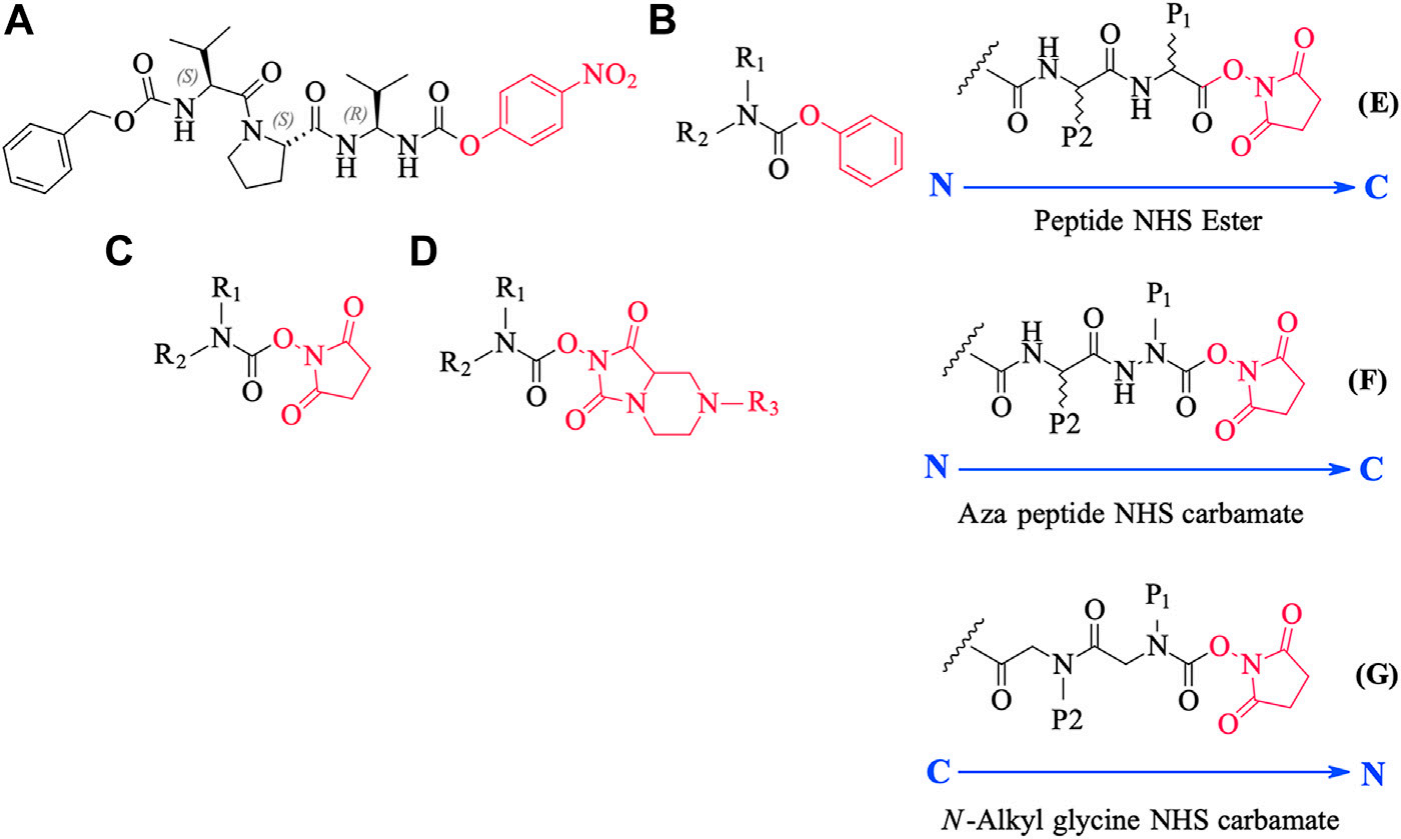
Chemical structures of various carbamate chemotypes **(A–D)** and generalised structures of a peptide NHS ester **(E)**, azapeptide NHS carbamate **(F)** and *N*-alkyl glycine NHS carbamate **(G)**. The blue arrow shows the direction of the amide bonds in each compound type.

We conceived of this ABP chemotype since previous studies had demonstrated that peptides containing activated carbamate derivatives of C-terminal gem diamines at P1 (Figure 10, **structure A**), functioned as irreversible inactivators of neutrophil elastase (Gilmore et al., 2006) and activated carbamates such as phenyl, *N*-hydroxy succinimide, and hydantoin derivatives of small molecule amines (structures **B** through **D**, respectively, in Figure 10), functioned as effective ABPs for the serine hydrolases (Chang et al., 2013; Niphakis et al., 2013; Cognetta et al., 2015; Otrubova et al., 2019).

**FIGURE 11:**
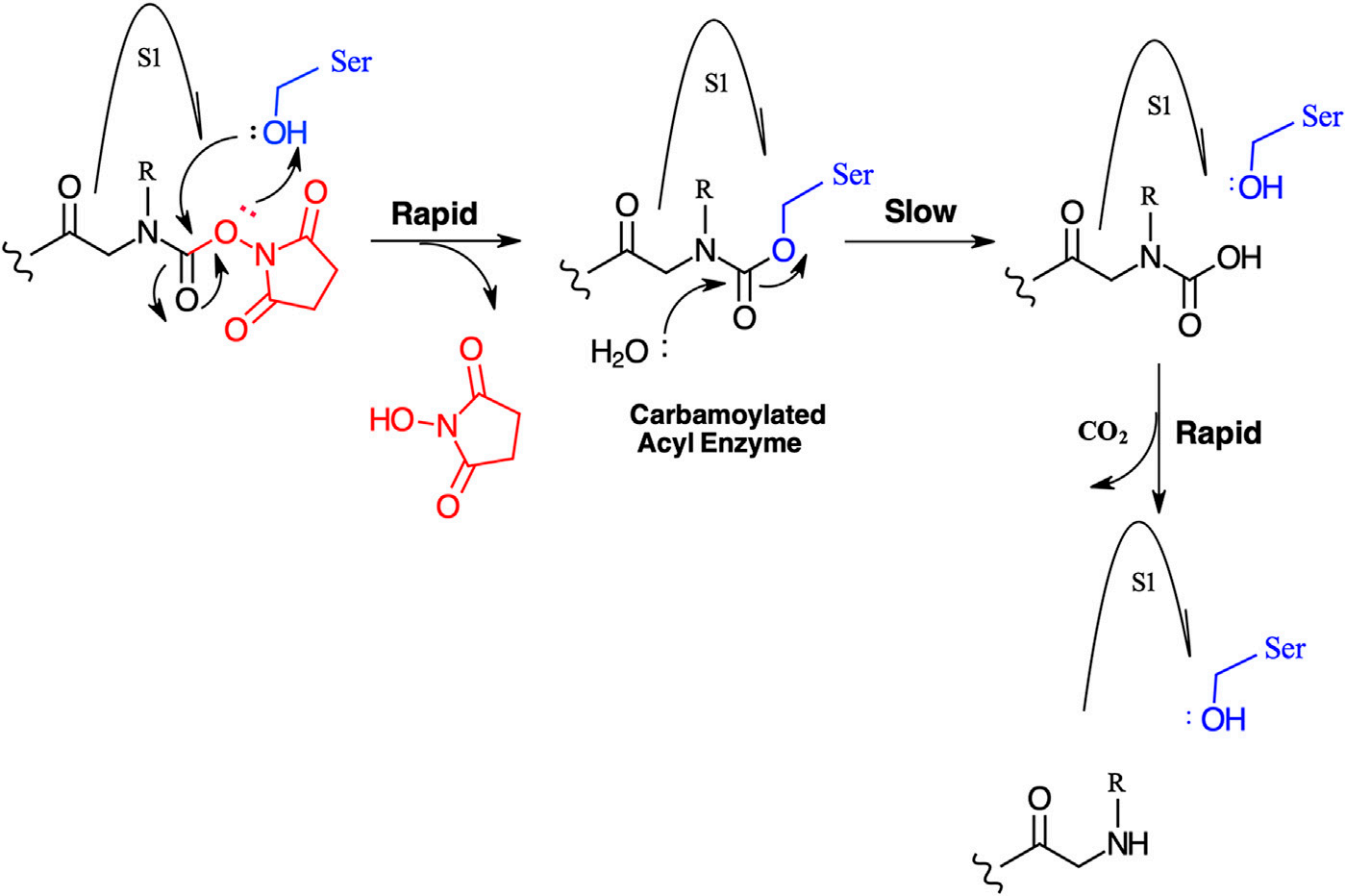
Postulated mechanism for the inhibition of serine proteases by *N*-alkyl glycine NHS carbamates.

We have yet to formally establish an inhibitory mechanism of action for these N-alkyl glycine carbamates. However, reflection on the similarity of the generalized structures for a peptide NHS ester, aza-peptide NHS carbamate, and N-alkyl glycine NHS carbamate (structures **E**, **F,** and **G**, respectively in Figure 10), provides a basis for developing a likely mechanistic hypothesis. Since aza-peptide carbamates are known to form covalent, long-lived carbamoylated enzymes with serine proteases (Powers and Gupton, 1977; Nardini et al., 1996), it is likely that the N-alkyl glycine carbamates operate via a similar mechanism (see Figure 11). Direct observation of the formation of this postulated carbamolyated-enzyme intermediate was not confirmed. However, the kinetic analysis of the inhibition processes performed on the range of the newly described *N*-alkyl glycine carbamates against their respective target serine proteases fits with irreversible inhibition models (Tian and Tsou, 1982; Walker and Elmore, 1984; Tsou 1988). Additionally, the ability to detect a broad range of SP–ABP complexes following the denaturing conditions of SDS-PAGE resolution would rule out the formation of exclusively non-covalent complexes between the target SP and cognate ABP and lends credence to our proposed inhibitory mechanism. Finally, the *in silico* docking studies discussed above also support our thesis that it is likely that these peptidyl *N*-alkyl glycine NHS-carbamates form carbamoylated–enzyme complexes with the active site serine residue of their target SPs.

One driver for undertaking the development of peptidyl *N*-alkyl glycine NHS-carbamates is their ease of synthesis, which is in direct contrast to the preparation of the majority of the commonly employed serine protease ABP chemotypes (phosphonate, phosphinate, and *iso*-coumarin), which requires considerable synthetic chemistry acumen. In essence, the *N*-alkyl glycine carbamates detailed in the present study and the *N*-alkyl glycine arginine mimetics described in our previous publication (Ferguson et al., 2022) can be prepared by the exclusive use of solid-phase synthesis methods. This makes them potentially more widely accessible to groups with research interests in serine protease biology and with limited expertise in organic synthesis but with access to peptide synthesis core facilities. It is anticipated that peptidyl *N*-alkyl glycine NHS-carbamates will find increasing application as serine protease ABPs because of their ease of synthesis, apparent lack of inhibitory activity against the mechanistically similar cysteine protease class, and demonstrable utility for profiling for serine proteases in complex biological samples, such as CF Sol (present study) and cockroach extracts (Ferguson et al., 2022).

## Data Availability

The original contributions presented in the study are included in the article/Supplementary Material, and further inquiries can be directed to the corresponding author.
