# Supplementary material for: Novel inhibitors and activity-based probes targeting serine proteases
Source: Front Chem. 2022 Sep 28;10:1006618. doi: 10.3389/fchem.2022.1006618 (PMC9555310; doi:10.3389/fchem.2022.1006618)
Supplement: Supplementary file 1 [file DataSheet1.DOCX]

**Experimental**

**Materials**

1. **Materials for Synthesis**

ESI-MS analysis was carried out by ASEPT (Queen’s University Belfast). HPLC analysis of final compounds was carried out on an Agilent Technologies 1260 Infinity machine using a Waters Atlantis C18 5 μm, 4.6 x 150m column. A two-phase solvent system was used, A) 0.05% (v/v) TFA in water and B) 0.05% (v/v) TFA in acetonitrile. A linear gradient elution system was implemented at 1 ml/min from 0% B) to 90% B) over 45 minutes, held for a further 10 minutes, then back to 0% B) over 10 minutes and held for a final 10 minutes. A UV detector was used to monitor absorbance at λ = 216 nm.

All reagents and solvents were supplied by Sigma-Aldrich unless otherwise indicated. All standard Fmoc-protected amino acids and HBTU were supplied by Activotec. Coupling agents HCTU and HATU were supplied by Novabiochem. Fmoc-Rink Amide MBHA resin and Biotin-PEG NovaTag resin were supplied by NovaBiochem. DIPEA (Sigma-Aldrich) was purified by distillation over potassium hydroxide prior to use. Di-t*ert*-butyl decarbonate and trifluoroacetic acid were supplied by Fluorochem. Flash Chromatography purification was carried out using Silica Gel for Flash Chromatography supplied by Fluorochem. Fmoc-NArg-(Pbf)-OH, Fmoc-NLys-(Boc)-OH, Fmoc-NLeu-OH and Fmoc-NPhe-OH were supplied by PolyPeptide Laboratories.

1. **Materials for Protease Assays**

Fluorogenic enzyme assays were carried out on a FLUOstar OPTIMA spectro-fluorimeter (BMG Labtech) with analysis performed on MARS Data Analysis Software (BMG Labtech). Black 96-well microtitre plates were supplied by Thermo Scientific. Trypsin from porcine pancreas, plasmin from human plasma and chymotrypsin from bovine pancreas were obtained from Sigma-Aldrich. Thrombin and Cathepsin B were supplied by R and D Systems. Human Neutrophil Elastase was supplied by Calbiochem. Fluorogenic substrates Z-Gly-Gly-Arg-NHMec, Succinyl-Ala-Ala-Pro-Phe-NHMec and MeO-Succinyl-Ala-Ala-Pro-Val-NHMec were supplied by Sigma-Aldrich. Boc-Val-Pro-Arg-NHMec was obtained from R and D Systems and Boc-Val-Leu-Lys-NHMec was supplied by PeptaNova. Phosphate Buffered Saline tablets were acquired from Sigma- Aldrich.

1. **Materials for Electrophoresis and Western Blotting**

SDS-PAGE was carried out using NuPAGE Novex 4-12% bis-Tris Protein Gels 1.0 mm using a PowerEase 500 power supply with a SeeBlue Plus2 Pre-stained Protein Standard as a reference ladder, all supplied by Invitrogen. Rhodamine-X azide, biotin azide and Copper (II)-TBTA complex were supplied by Lumiprobe. Western blotting was achieved with an X- Cell II Blot Module (Invitrogen) unto an Amersham Hybond ECL Nitrocellulose membrane (GE Healthcare). Luminata Forte Western HRP substrate was supplied by EMD Millipore. Streptavidin-HRP was supplied by Vector Laboratories. BSA, Tween 20, Tris and sodium chloride were supplied by Sigma-Aldrich. Gels and blots were visualised using a UVP bioimaging system.

1. **Materials for ELISA measurement of NE in CF Sol.**

The amount of active NE in CF sol was determined using the ProteaseTag® Active Neutrophil Elastase Immunoassay, obtained from Proaxsis Ltd. The assay was performed exactly according to the manufacturer’s instructions. ELISAs were carried out on 96-well Streptavidin-Immobilizer plates (Nunc, Thermo Fisher Scientific). Plate washing was carried out using a Biotek ELx50 plate washer (Biotek).

**Methods**

**General Peptide Synthesis Protocols**

1. **Resin Preparation/Swelling**

Dry resin was weighed out sufficient for the required synthesis scale and placed in a sintered glass funnel, in a manual bubbling apparatus. Dimethyl formamide (DMF) approximately 20 mL was added, and the resin was agitated gently, using nitrogen gas, for 30 minutes.

1. **Fmoc Deprotection**

Method A

Removal of the Fmoc amino protecting group was achieved by addition of a solution of piperidine in DMF (20% v/v), to the resin. The mixture was agitated, using nitrogen gas, for 30 minutes, until Kaiser analysis indicated a positive result.

Method B

Alternatively, Fmoc deprotection was achieved by addition of 3% 1,8-Diazabicyclo-(5.4.0)-undec-7-ene (DBU) in DMF (3% v/v), for 10 minutes. This was repeated a further two times to ensure complete deprotection.

1. **Resin washing**

Following Fmoc-deprotection and amino acid coupling, the peptide-resin was washed thoroughly with DMF (3 x 5 minutes) and Dichloromethane (DCM) (3 x 5 minutes). Prior to cleavage of the final peptide sequences, the peptide resin was washed with DMF (3 x 5 minutes) followed by DCM (5 x 5 minutes), to ensure complete removal of DMF. The peptide-resin was then dried overnight, *in vacuo*, over silica gel.

1. **Coupling of amino acid and *N*-alkyl glycine building blocks**

A solution of *N*-a-Fmoc-protected amino acid (3-4 eq.), or *N*-a-Fmoc-protected *N*-alkyl glycine building block (3-4 eq.) and HATU (3-4 eq.) were dissolved in approximately 20 mL of DMF or *N*-Methyl-pyrrolidone (NMP). To this was added DIPEA (3-4 eq.) and the whole solution was then added to the deprotected resin. The reaction mixture was agitated gently with nitrogen gas until Kaiser analysis indicated complete coupling (most coupling reactions went to completion within 2-3 hours).

1. **Cleavage of target peptides and Side-Chain Deprotection**

Simultaneous cleavage of the target peptides from the resin support and side-chain deprotection were carried with the use of trifluoroacetic acid. To the dried peptide-resin was added a cleavage cocktail of TFA/TIPs/H2O (95:2.5:2.5, 10 ml/g resin). The cleavage mixture was stirred gently at room temperature for two hours, after which time the cleaved resin was removed by filtration and washed twice with DCM. The filtrate was concentrated under reduced pressure to approximately a 1 ml solution, which was added to ice-cold diethyl ether (50 ml), followed by overnight storage at -20oC. The precipitated peptide was collected by centrifugation and washed with further diethyl ether (3 x 20 ml) the peptide was then dried overnight in a desiccator over KOH/silica.

1. **Determination of coupling effectiveness and extent of modification of resin-bound amines.**

Kaiser test for Primary Amines

A small sample of resin was washed with DMF and methanol. To this was then added a few drops of each of the following reagents sequentially:

a)  ninhydrin (1 g) in ethanol (20 ml),

b)  phenol (40 g) in ethanol (20 ml) and

c)  potassium cyanide (200 μl of 1 mM aqueous solution, made up to 10 ml with pyridine).

The suspension was then heated, at 100^o^C, for 10 minutes. An intense blue colour indicated the presence of free amino groups, whereas no colour change indicated the absence of free amino groups. The presence of secondary amines such as proline results in formation of red colouration of the resin beads.

Chloranil test for Secondary Amines

A small sample of resin was transferred to an Eppendorf tube and washed with methanol and DMF by brief centrifugation. One drop of acetaldehyde solution (2% (v/v) in DMF) and one drop of chloranil solution (2% (w/v) in DMF) was added. The resin was left to sit for ten minutes, after which time the colour was observed. Blue-green beads indicated presence of secondary amines, whereas no colour change suggested the absence of secondary amines.

**Synthesis of peptidyl N-Alkyl glycine *N*-Hydroxy succinimidyl carbamates**

**NAP830 Succinimidoyl-CO-NLys-Gly-Gly-PEG-Biotin**

Synthesis of this peptide was carried out using Biotin-PEG NovaTag resin (Novabiochem) (104 mg, 0.05 mmol). The resin was swollen and deprotected using the standard protocols outlined above. To the washed resin was added a solution containing Fmoc-NLys-(Boc)-OH (PolyPeptide Laboratories) (194.6 mg, 0.3 mmol), HOBt (45.96 mg, 0.3 mmol) and DIC (46.5 μl, 0.3 mmol) in NMP, followed by addition of DIPEA (52.3 μl, 0.3 mmol). Upon completion of the coupling reaction, following overnight agitation, the resin was washed and deprotected as previously described. The resin was suspended in anhydrous DCM/DMF (1:1), to which was added *N, N’*-disuccinimidyl carbonate (DSC) (76.9 mg, 0.3 mmol) and 2,6-lutidine (35 μl, 0.3 mmol). The suspension was agitated for 6 hours until complete reaction had occurred as indicated by a negative chloranil result. The resin was washed with anhydrous DMF (x 1) and DCM (x 3) and dried overnight *in vacu*o. Cleavage of the desired peptide was carried out with addition of TFA in DCM (7:3) for 2 hr, followed by filtration and concentration under reduced pressure. The peptide was precipitated in diethyl ether, collected by centrifugation, and washed with further diethyl ether, before being dried in vacuo to give the title compound as an off-white solid of the TFA salt (67 mg, quantitative yield). ESI-MS m/z 830.42 [M + H+] (calculated C35H59N9O12S: 829.40).

**NAP849 Succinimidoyl-CO-NPhe-Gly-Gly-PEG-Biotin**

Peptide was synthesised on a 0.05 mmol scale as described for NAP830, with the exception that Fmoc-NPhe-OH (PolyPeptide Laboratories) was used as the *N*-Alkyl glycine building block. Cleavage was carried out with TFA/DCM (9:1) for 2 hours to give the title compound as a pale red oil (69.9 mg, quantitative yield). ESI-MS m/z 708.38 (- Succinimide) [M + H^+^]; 866.41 [M + NH_4_^+^] (calculated C38H56N8O12S: 848.37).

**NAP800 Succinimidoyl-CO-NVal-Gly-Gly-PEG-Biotin**

This ABP was synthesised on a 0.05 mmol scale. was using the submonomer approach, essentially according to a previously reported method (Tran *et al.* 2011) (**Figure 3B**). Consequently, after derivatisation of the Biotin-PEG NovaTag resin with the two glycine residues (following the same protocols outlined for NAP830 above), the terminal glycine residue was deprotected (Fmoc-group removal) and the resin reacted with a solution containing bromoacetic acid (69.5 mg, 0.5 mmol) and DIC (72 μl, 0.465 mmol) in DMF (3 ml), with agitation, for 30 minutes. The solution was removed by filtration and replaced with a freshly made solution. This was repeated for a total of four times until Kaiser analysis indicated complete bromo-acetylation of the resin. The resin was washed once with DMF followed by addition of NMP (2 ml) and *di*-isopropylamine (1 ml), and the resin was then agitated for 3 hours. The NHS carbamate was then formed through reaction with DSC as described previously. Cleavage was performed by addition of TFA/DCM (9:1) for 2 hr and following filtration and removal of solvents *in vacuo,* the compound was isolated as a pale red oil (77.3 mg, quantitative yield). ESI-MS m/z 640.59 (- Succinimide) [M + NH_4_^+^]; 818.41 [M + NH_4_^+^] (calculated C34H56N8O12S: 800.37).

**NAP962 Succinimidoyl-CO-NLys-NPhe-NVal-PEG-Biotin**

Biotin-PEG NovaTag resin (Novabiochem) (0.05 mmol) was swollen in DMF for 30 minutes followed by deprotection under standard conditions. Following Fmoc-deprotection, an NVal residue was introduced by the submonomer bromo-acetylation-substitution method described for NVP800. This was followed by subsequent coupling and deprotection of Fmoc-NPhe-OH and Fmoc-NLys-(Boc)-OH. The NHS carbamate was formed as described for NAP830, by reaction with DSC. Following treatment with TFA/DCM (8:2) for 2 hr, the cleaved resin was removed by filtration, washed with DCM, and the filtrate concentrated under reduced pressure to give the title compound as a trifluoroacetate salt (110.9 mg, quantitative yield). ESI-MS m/z 481.75 [M + 2H^+^]/2; 962.50 [M + H^+^] (calculated C45H71N9O12S: 961.49).

**NAP559 Succinimidoyl-CO-NArg-D-Pro-NPhe-NH_2_**

NAP559 was synthesised on MBHA Rink Amide resin (139 mg, 0.05 mmol). The resin was swollen in DMF (15 min) and the resin-bound Fmoc protecting removed, using standard protocols. The target peptide was synthesised using standard methods of coupling and deprotection, employing sequentially Fmoc-NPhe-OH, Fmoc-D-Pro-OH and Fmoc-NArg-(Pbf)-OH. The NHS carbamate was formed through reaction with DSC as described above. Peptide release from the resin and side-chain protecting group cleavage were achieved by reaction with a solution of TFA/TIPS/DCM (95:2.5:.5), for 2 hr, followed by filtration and concentration under reduced pressure. The residue was precipitated in diethyl ether and washed further with diethyl ether (3x). The title compound was isolated as the trifluoroacetate salt (25.2 mg, 75%). ESI-MS m/z 462.20 (- Succinimide) [M + NH_4_^+^]/2; 559.25 [M + H^+^]; 577.27 [M + NH_4_^+^] (calculated C25H34N8O7: 558.25).

**NAP533 Succinimidoyl-CO-NLys-NPhe-Val-NH_2_**

NAP553 was synthesised on MBHA Rink Amide resin (278 mg, 0.1 mmol). The resin was swollen in DMF (15 min) and the resin-bound Fmoc protecting removed, using standard protocols. The target peptide was synthesised using standard methods of coupling and deprotection, employing sequentially Fmoc-Val-OH and Fmoc-NPhe-OH and Fmoc-NLys-(Boc)-OH. The NHS carbamate was formed through reaction with DSC as described above. Peptide release from the resin and side-chain protecting group cleavage were achieved by reaction with a TFA (5 mL), for 2hr, followed by filtration and removal of the volatiles under reduced pressure to give the title compound as a trifluoroacetate salt (17.3 mg, 81%). ESI-MS m/z 533.27 [M + H^+^] (calculated C25H36N6O7: 532.26).

**Inhibition studies**

Protease inactivation by each of the compounds under study was evaluated using a fluorogenic enzyme assays. Protease-substrate combinations used as detailed in **Section 2**. Inhibitors were made up as 10 mM stock solutions in either DMF or DMSO, then diluted with PBS (pH 7.4) to give appropriate working concentrations. A final volume of 100 μL was used per well. Substrate (50 μM final concentration) was added to a black 96-well microtitre plate (Thermo Scientific), in duplicate, followed by addition of inhibitor, in such a manner to give the required final concentration. Control wells consisted of substrate with the absence of inhibitor and blank wells were composed of substrate and buffer. Enzyme was added to all wells except the blank wells, to give a final volume of 100 μl containing the amount of protease specified in Table 6.1. Increase in fluorescence was recorded using a FLUOstar OPTIMA spectrofluorometer (BMG Labtech), excitation 380 nm and emission 460 nm, for 61 cycles (60 seconds/cycle), with results analysed using MARS Data Analysis Software (BMG Labtech).

For assays requiring a pre-incubation step enzyme and inhibitor in assay buffer were incubated together, at 37^o^C, for 2 min, with gentle agitation. The reaction mixture was then aliquoted (50 μL) in duplicate on a black 96-well microtitre plate followed by addition of substrate to make a final concentration of 50 μM in a total volume of 100 μL. The fluorescence production was then measured as described for the previous procedure.

Assays with Cathepsin B required a pre-activation step of the protease prior to use in the fluorogenic enzyme assays. Cathepsin B was diluted to 10 μg/ml in activation buffer (0.1 M MES, pH 6.1, containing 1 mM EDTA and 2 mM DTT) and left to stand at room temperature for 15 minutes. Activated Cathepsin B was then diluted (1:50 to give 10 ng/50 μl) with assay buffer (activation buffer minus the DTT) prior to use as described previously.

**Electrophoretic and Western blotting methods**

SDS-PAGE

Protein samples to be resolved by SDS-PAGE under reducing conditions were diluted with 5x Reducing Treatment Buffer, containing 0.5 M Tris pH 6.8 (1.25 ml), SDS (1 g), glycerol (4 ml), 2-mercaptoethanol (2.5 ml), DDW (2.25 ml) and bromophenol blue (to colour). Samples were heated at 95oC for 10 minutes before being loaded into a NuPAGE Novex 4- 12% (w/v) bis-Tris Protein Gels 1.0 mm (Invitrogen). SeeBlue Plus2 Pre-stained Protein Standard (Invitrogen) (5 μl) was added as a reference ladder. Following loading, gels were ran using a PowerEase 500 system (Invitrogen) set at 200 V, 120 mA for 35 minutes, in SDS- MES running buffer (Invitrogen). Following electrophoretic separation, gels were removed and used for Western blotting.

Western Blotting

Protein transfer from an acrylamide gel unto an Amersham Hybond ECL Nitrocellulose membrane (GE Healthcare) was achieved with the use of a X-Cell II Blot Module (Invitrogen). The gel and membrane were sandwiched between filter paper and sponges soaked in transfer buffer (25 mM Tris, 192 mM glycine, 10% (v/v) MeOH), placed in the blotting module and ran utilising a PowerEase 500 (Invitrogen) power pack set at 25 V, 125 mA for 1.5 hours.

Following transfer, the extra protein binding sites on the membrane were blocked by addition of 3% (w/v) BSA in TBST (20 mM Tris, 137 mM NaCl, 1% (v/v) Tween 20, pH 7.6) and agitated gently at room temperature for 30 minutes. The biotin-labelled proteases were then detected by addition of Streptavidin-HRP (Vector Laboratories) in 3% (w/v) BSA in TBST at a concentration of 1:20000, for 1 hour at room temperature. The membrane was then washed with TBST (3 x 5 minutes), followed by addition of Luminata Forte Western HRP substrate (EMD Millipore) and visualised using a UVP bio-imager.

**Supplemental data**

**ESI-MS Spectra**

**NAP559 Succinimidoyl-CO-NArg-D-Pro-NPhe-NH_2_**

**NAP533 Succinimidoyl-CO-NLys-NPhe-Val-NH_2_**

**NAP849 Succinimidoyl-CO-NPhe-Gly-Gly-PEG-Biotin**

**Table ST1.** Kinetic constants for the inhibition of thrombin by NAP559.

| Inhibitor | K_i_ (M) | k_3_ (min^-1^) | k_3_ / K_i_ (M^-1^ min^-1^) |
| --- | --- | --- | --- |
| NAP559 (n=2) | 5.67 (± 0.9) x 10^-6^ | 0.6 (± 0.068) | 1.1 (± 0.3) x 10^5^ |

Values shown as Mean ± SD.


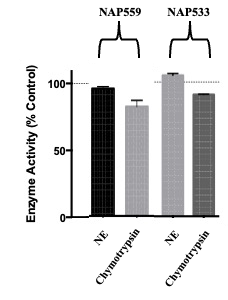


**Figure ST1**. Results of “off-target” studies. NAP559 and NAP533 were tested against chymotrypsin, and NE. Samples (approx. 0.01 mg) of each protease were incubated with the indicated concentrations of test compound for 2 min, in PBS, pH 7.4, at 37^o^C. Aliquots (approx. 10 μL) of each incubation sample were then added to a fresh sample (990 μL) of PBS containing the appropriate substrate for each protease, to measure their residual activity. Controls incubations were performed in the absence of test compound, but in the presence of an appropriate volume of DMF vehicle used to prepare stock solutions of each inhibitor under study.

**Table ST1**. Kinetic constants for the inhibition of thrombin by NAP559.

| K_i_ (M) | k_3_ (min^-1^) | k_3_ / K_i_ (M^-1^.min^-1^) |
| --- | --- | --- |
| 5.7 (+ 0.9) x 10^-6^ | 0.5 + 0.006 | 1.1 (+ 0.3) x 10^5^ |

Values shown as Mean ± SD (n=2). Assays were carried out in PBS, pH 7.4, at 37^o^C, in the presence of a range of inhibitor concentrations (0.25μM–50μM) and a fixed concentration (50μM) of the substrate Boc-Val-Pro-Arg-NHMec.
